# *TAS2R38* is a novel modifier gene in patients with cystic fibrosis

**DOI:** 10.1038/s41598-020-62747-9

**Published:** 2020-04-02

**Authors:** Alice Castaldo, Gustavo Cernera, Paola Iacotucci, Chiara Cimbalo, Monica Gelzo, Marika Comegna, Antonella Miriam Di Lullo, Antonella Tosco, Vincenzo Carnovale, Valeria Raia, Felice Amato

**Affiliations:** 1 Dipartimento di Scienze Mediche Traslazionali, Sezione di Pediatria, Università di Napoli Federico II, CRR Fibrosi Cistica del Bambino, Naples, Italy; 2 Dipartimento di Sanità Pubblica, Università di Napoli Federico II, Naples, Italy; 3 Dipartimento di Medicina Molecolare e Biotecnologie Mediche, Università di Napoli Federico II, Naples, Italy; 4 CEINGE Biotecnologie avanzate, s.c.a.r.l., Naples, Italy; 5 Dipartimento di Scienze Mediche Traslazionali, Sezione di Geriatria, Università di Napoli Federico II, CRR Fibrosi Cistica dell’Adulto, Naples, Italy; 6 Dipartimento di Neuroscienze, Sezione di ORL, Università di Napoli Federico II, Naples, Italy

**Keywords:** Genetics, Diseases

## Abstract

The clinical manifestation of cystic fibrosis (CF) is heterogeneous also in patients with the same *cystic fibrosis transmembrane regulator (CFTR)* genotype and in affected sibling pairs. Other genes, inherited independently of *CFTR*, may modulate the clinical manifestation and complications of patients with CF, including the severity of chronic sinonasal disease and the occurrence of chronic *Pseudomonas aeruginosa* colonization. The *T2R38* gene encodes a taste receptor and recently its functionality was related to the occurrence of sinonasal diseases and upper respiratory infections. We assessed the *T2R38* genotype in 210 patients with CF and in 95 controls, relating the genotype to the severity of sinonasal disease and to the occurrence of *P. aeruginosa* pulmonary colonization. The frequency of the PAV allele i.e., the allele associated with the high functionality of the T2R38 protein, was significantly lower in i) CF patients with nasal polyposis requiring surgery, especially in patients who developed the complication before 14 years of age; and ii) in CF patients with chronic pulmonary colonization by *P. aeruginosa*, especially in patients who were colonized before 14 years of age, than in control subjects. These data suggest a role for *T2R38* as a novel modifier gene of sinonasal disease severity and of pulmonary *P. aeruginosa* colonization in patients with CF.

## Introduction

Cystic fibrosis is a severe autosomal recessive disease due to mutations in the *cystic fibrosis transmembrane regulator* (*CFTR*) gene. The clinical manifestation of cystic fibrosis (CF) is highly heterogeneous also depending on the functional effect of the *CFTR* genotype^1,2^. However, patients with the same *CFTR* genotype may display a clinical discordance^3^ and a percentage of sibling pairs affected by CF display a discordant clinical manifestation^3,4^ reinforcing the concept that complex alleles (i.e., additional mutations on the same allele^5^), non-coding regions of *CFTR*^6,7^ or other genes, inherited independently of *CFTR*, modulate the clinical manifestation and complications of patients with CF^8^. Patients with CF frequently show different degrees of chronic rhinosinusitis^9,10^ with poor perception of sinonasal symptoms^11^ and pulmonary colonization by *Pseudomonas aeruginosa* and other opportunistic bacteria^12,13^. Chronic rhinosinusitis is common in patients affected by CF and it causes smell alterations^14^ that may impair taste and cause alterations in nutrition, thus, potentially impacting overall therapy outcome^15^. A percentage of patients develop nasal turbinate hypertrophy (NTH) or nasal polyposis (NP)^10^. However, according to the US CFF registry^16^, only 2–3% of patients every year require surgery for NP, which is considered the final, most advanced step of sinonasal disease. It is still unclear why only a small subgroup of patients with CF progress to such phase of the sinonasal disease^17–20^, even though the severity of *CFTR* mutations as well as previous sinus surgery may predict an increased risk^11^. Interestingly, in a recent study on 101 CF sibling-pairs^4^ we found NP requiring surgery in both the siblings of 13 pairs and in only one sibling from 12 sibling pairs. The poor correlation of NP to the *CFTR* genotype and to other clinical manifestations such as the pancreatic status and the severity of lung disease suggest that modifier genes play a role in determining nasal polyposis^21^.

*P. aeruginosa* is an opportunistic pathogen that frequently infects the lungs of patients with CF contributing to the decline in pulmonary function. *P. aeruginosa* expresses a series of specific virulence factors and, through adaptive mutations and antimicrobial resistance, causes chronic colonization via the development of biofilms^22^. In our study on CF sibling pairs^4^, we found chronic colonization by *P. aeruginosa* in 92/208 (44.2%) patients with CF and 21/101 cases in which only one sibling was colonized; 11/21 of these patients lived in the same environment suggesting that the environment has a limited role in *P. aeruginosa* colonization. Modifier genes, inherited independently by *CFTR*, may predispose to colonization of *P. aeruginosa*^23^.

Bitter receptors are G protein-coupled proteins that detect bitter compounds ingested with the diet^24^. The *T2R38* gene, originally identified in type II taste receptor cells of the tongue, encodes one of these proteins, which exerts a main role as bitter taste receptors with the aim of protecting the individual against the ingestion of toxic substances present in spoiled foods. Among these substances, the receptor recognizes bacterial products such as acyl-homoserine lactones secreted by several gram-negative bacteria including *P. aeruginosa*, and it is now known that T2R38 and other bitter and sweet taste receptors are widely expressed by the upper respiratory tract ciliated cells and by solitary nose chemosensory cells^25,26^. These receptors, once activated by bacterial products, modulate ciliary beat frequency, promote the production of NO, and stimulate the release of immune peptides^27^. Such responses are strongly reduced in subjects who carry the nonfunctional “AVI” allele, while they are more effective in subjects carrying the “PAV” allele^25,27^ of the *T2R38* gene. Furthermore, this polymorphism produces changes in the amino acid residues at positions 49, 262, and 296, generating two common alleles: the functional taster allele encodes proline, alanine, and valine (PAV); and the nonfunctional non-taster allele encodes alanine, valine, and isoleucine (AVI). These two common alleles generate three common genotypes (i.e., PAV/PAV, PAV/AVI, and AVI/AVI). Various studies have described the increased occurrence of sinonasal diseases and upper respiratory infections in patients with altered T2R38 activity, including in patients with CF^28–32^.

## Results

### Correlation between *TAS2R38* and sinonasal complications

**Figure 1:**
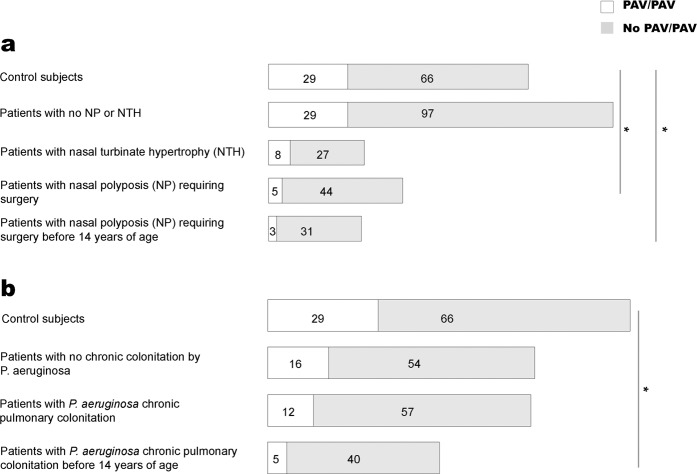
Frequency of the PAV/PAV homozygous genotype of the *TAS2R38* gene in (**a**) patients with CF and different degrees of sinonasal disease and in (**b**) patients with CF and *P. aeruginosa* chronic pulmonary colonization; *p < 0.01.

As shown in Table 1, we evaluated the frequency of the PAV allele and, as shown in Fig. 1a, we evaluated the frequency of the PAV homozygous genotype of the *TAS2R38* gene in various subgroups of CF patients with different levels of sinonasal complications, in comparison with those of control subjects. The PAV allele was found in 33/98 (33.7%) alleles from CF patients with NP requiring surgery and these figures were significantly lower than those for control subjects (p < 0.01). This frequency was even lower in CF patients who developed NP requiring surgery before 14 years of age (18/68 alleles, 26.4%) and again significantly lower than that in control subjects (p < 0.01). However, the allele frequency of PAV was not significantly different between CF patients with NTH and control subjects. The same was true for CF patients with no NP or NTH.

**Table 1:** Allele frequency of PAV in different subgroups of patients with CF and in control subjects.

Group	PAV allele
Control subjects	103/190 (54.2%)
CF with no NP - NTH	126/252 (50.0%)
CF with NTH	33/70 (47.1%)
CF with NP requiring surgery	33/98 (33.7%)*
CF with NP requiring surgery <14 yr old	18/68 (26.4%)*
CF with no Pa CC	67/140 (47.9%)
CF with Pa CC	53/138 (38.4%)*
CF with Pa CC < 14 yr old	28/90 (31.1%)*

NP: nasal polyposis; NTH: nasal turbinate hypertrophy; Pa: *Pseudomonas aeruginosa*; CC: chronic colonization.. *p < 0.01 as compared to control subjects.

These results were partially mirrored by the comparison of the frequency of the PAV homozygous genotype (Fig. 1a). It was significantly less frequent in patients with NP requiring surgery (5/49, 10.2%) than in control subjects (29/95, 30.5%; p < 0.01) and even lower in CF patients who developed NP requiring surgery before 14 years of age (3/34, 8.8%); the difference with control subjects was significant (p < 0.01). Again, the frequency of the PAV homozygous genotype did not differ between either CF patients with NTH and patients with no NP or NTH compared to that for control subjects.

### Correlation between *TAS2R38* and *P. aeruginosa* chronic colonization

Table 1 shows that the frequency of the PAV allele of the TAS2R38 gene was significantly lower in CF patients with P. aeruginosa chronic colonization (CC) (53/138, 38.4%) than in control subjects (p < 0.01), and it was even lower in patients colonized before 14 years of age (28/90, 31.1%), which was significantly lower (p < 0.01) than in control subjects. However, the frequency of the PAV allele did not differ between CF patients with no P. aeruginosa CC and control subjects. Again, the comparison of the frequency of the homozygous PAV allele (Fig. 1b) mirrored the results, being lower (though not significantly) in patients with P. aeruginosa CC (12/69 patients, 17.4%) than in control subjects, and even lower in CF patients colonized before 14 years of age (5/45, 11.1%), which was significantly lower than in control subjects (p < 0.01). No significant difference was observed between CF patients with no CC and control subjects.

### *TAS2R38* genotype in CF sibling pairs discordant for *P. aeruginosa* CC or NP requiring surgery

We included 34 pairs and two triplets of siblings affected by CF in the study. In 23 pairs of siblings we observed concordance between siblings of each pair for *P. aeruginosa* CC and NP requiring surgery. In particular, in 12 sibling pairs both members were colonized and in 11 pairs of siblings both members were not colonized; in 4 sibling pairs, both members experienced NP requiring surgery, and in 19 sibling pairs, both members did not experience such complications. In these 23 sibling pairs the PAV genotype was concordant (data not shown). As shown in Table 2, in 9 pairs and in two triplets of CF siblings we observed discordance between siblings for *P. aeruginosa* CC (5 sibling pairs) or NP requiring surgery (6 cases). In 7/11 discordant sibling pairs, both members had the same *TAS2R38* genotype. In 4/11 pairs (pairs # 2, 4, 5 and 6) we observed a different genotype and in all cases the PAV allele was present (homozygous or heterozygous) in the member not affected by the complication.

**Table 2:** *Pseudomonas aeruginosa* chronic colonization (CC), nasal polyposis (NP) requiring surgery and *TAS2R38* genotype in pairs of siblings affected by CF and discordant for CC and NP.

Sibling pair	*P. aeruginosa* CC	NP requiring surgery	*TAS2R38* genotype
1A	NO	YES	AVI/PAV
1B	NO	NO	AVI/PAV
2A	YES	YES	AVI/AVI
2B	YES	NO	AVI/PAV
3A	NO	NO	AVI/AVI
3B	YES	NO	AVI/AVI
3C	YES	NO	AVI/AVI
4A	NO	YES	AVI/AAV
4B	NO	NO	PAV/PAV
5A	YES	NO	AAV/PAV
5B	NO	NO	PAV/PAV
6A	YES	NO	AVI/AVI
6B	NO	NO	PAV/PAV
7A	NO	YES	AVI/PAV
7B	NO	NO	AVI/PAV
8A	YES	NO	AVI/PAV
8B	NO	NO	AVI/PAV
9A	NO	NO	AVI/AVI
9B	YES	NO	AVI/AVI
10A	NO	YES	AVI/PAV
10B	NO	NO	AVI/PAV
11A	NO	NO	AVI/AVI
11B	NO	YES	AVI/AVI
11C	NO	NO	AVI/AVI

## Discussion

Our study, performed on the largest population of patients studied so far in this field, demonstrates that the frequency of the PAV allele of the *TAS2R38* gene, i.e., the allele associated with the high functionality of the protein, was significantly reduced in CF patients with NP requiring surgery, the most advanced phase of CF sinonasal complications^18^. This finding confirms the relationship between the altered function of the TAS2R38 protein and the risk of upper airway infections^31^ and chronic rhinosinusitis^28^. The frequency of the PAV allele of the *TAS2R38* gene was also significantly reduced in CF patients with chronic pulmonary colonization by *P. aeruginosa*, suggesting - for the first time - a role of the altered protein as a risk factor for lower respiratory infections.

Concerning the correlation between *TAS2R38* and sinonasal diseases, in 2013, a pilot study on 28 patients with chronic rhinosinusitis demonstrated that supertaster patients (i.e., those with the highest activity of the TAS2R38 receptor being homozygous for the PAV allele) had a reduced risk of progressing to a disease requiring surgery. The result was later confirmed with a higher number of cases^30^. The same group studied 49 patients with CF, demonstrating that the patients homozygous for the PAV allele had a less severe sinonasal disease based on the SNOT score^29^ and the same results were reported in 123 non-CF patients with rhinosinusitis^28^. A single study that included only 53 patients with chronic rhinosinusitis and 39 healthy controls did not find associations between *TAS2R38* genotypes and sinonasal disease^33^. Finally, a study by Cantone *et al*.^34^ on 100 subjects confirmed the inverse correlation between TAS2R38 functionality and the risk of developing sinonasal infections caused by gram-negative bacteria. The present study, which included 210 patients with CF and 95 control subjects, confirms that CF patients with both functional alleles of *TAS2R38* have a significantly reduced risk of developing to the most severe phase of sinonasal disease, i.e., NP requiring surgery. Such complications occur only in 2–3% of patients with CF per year and it is still unclear why only a small subset of patients will progress to this phase of sinonasal disease^16,18^. Regarding surgical treatment, endoscopic sinus surgery for management of paranasal sinus diseases in CF patients is undoubtedly a challenge for the surgeon^35^. In the literature, surgery is indicated when CRS does not respond to conservative maximal medical therapy and is performed in 20–60% of patients with CF^36^. This variable frequency of surgically treated patients mirrors the absence of a consensus on indications and timing for surgery. In fact, no universally accepted guidelines for the surgical management of CRS in CF patients are available^36^.

Interestingly, our data relate the *TAS2R38* genotype to the age of onset of NP requiring surgery, indicating that CF patients with nonfunctional alleles would develop NP requiring surgery at a younger age than CF patients with functional alleles. Thus, *TAS2R38* can be considered a novel CF modifier gene, that modulates the severity of sinonasal disease contributing to explaining the known discordance for sinonasal severity observed in CF patients with the same *CFTR* genotype and in some pairs of CF patients^4^.

In addition, we also found a clear relationship between the *TAS2R38* genotypes and chronic pulmonary colonization by *P. aeruginosa*. Our data indicate that CF patients with functional alleles of the gene (including some discordant sibling pairs) have a reduced risk of developing chronic colonization by *P.aeruginosa*, and have a decreased risk of developing colonization in childhood (i.e., before 14 years of age). There are no data so far with which compare our results. All the studies performed so far relate the *TAS2R38* genotype only to upper respiratory infections, while this report is the first study that investigated patients with pulmonary colonization. However, considering that *P. aeruginosa* is among the bacteria that produce large amounts of acyl-homoserine lactones (known activators of the TAS2R38 receptor) we may speculate on the role of the TAS2R38 protein in the lower respiratory tract. Thus, if future studies confirm the present results, *TAS2R38* would become a modifier gene that can impact *P. aeruginosa* infection in patients with CF. This result would help to improve management strategies to prevent *P. aeruginosa* colonization in CF patients with an elevated risk.

## Materials and Methods

### Study population

We recruited 210 patients with CF (105 males; age: 5–65 years; median: 20 years) followed at the Regional Cystic Fibrosis Reference Center. All subjects gave their informed consent for inclusion before they participated in the study. The study was conducted in accordance with the Declaration of Helsinki, and the protocol was approved by the Ethics Committee of Federico II University Hospital (244–2015). All the subjects (guardians in the case of minors) provided written informed consent to anonymously use their DNA samples and clinical data for research purposes Among these subjects, we studied 34 pairs and 2 triplets of siblings affected by CF. The data were anonymized. All patients met the criteria for the diagnosis of CF^37^. Airway colonization by *P. aeruginosa* was identified by sputum or oropharyngeal swab culture. Chronic colonization (CC) was defined according to the modified Leeds criteria^38^. The history of sinonasal disease including NTH and NP requiring surgery was evaluated by an otolaryngologist.

All subjects underwent the ENT (ear nose throat) examination by nasal endoscopy to assess the endoscopic appearance of the nose standardized by the Lund and Kennedy scale, which evaluates the clinical status of the nasal cavities (i.e., the presence of nasal polyps, oedema, discharge, scarring, and crusting)^14^. The ENT examination was performed with a 2.7-mm 30° rigid endoscope (Storz, Tuttlingen, Germany). All individuals enrolled in this study presented an endoscopic nasal score between 2 and 13, mean 5.65 ± 2.87, characterized by the presence of purulent discharge and diffuse oedema of the nasal mucosa in most of the cases. No sinonasal samplings (e.g. nasal lavage, nasal swabs) were performed because recent studies reported a relationship between upper and lower airway infection revealing concordant *S. aureus* and *P. aeruginosa* strains in upper and lower airways of the same patients^39^. No health related QoL (quality of life) assessment (e.g. SNOT-20 or SNOT-22) was performed because it is well known that SNOT-22 is frequently normal in patients with CF^9,14^.

In addition, as control we studied, 95 healthy subjects (38 males; age: 7–66 years; median: 18 years) who presented an endoscopic nasal score of 0 and who were characterized by the absence of any endoscopic sign of CRS.

### Sequencing analysis

Sweat chloride levels were tested according to the guidelines^40^ under quality control procedures^41^. In all patients, the *CFTR* genotype was defined by screening a panel of the most frequent point mutations^42^ and gene rearrangements^43^. Moreover, *CFTR* Sanger gene sequencing^44^ or NGS^45^ was performed when mutations were not detected in one or both alleles by first-level analysis. Furthermore, we analysed 7 intragenic *CFTR* short tandem repeats^46^ to verify that both members of three sibling-pairs carrying only one known mutation had the same *CFTR* genotype. DNA was extracted from blood samples with standard proteinase K digestion followed by phenol/chloroform extraction and ethanol precipitation. Following extraction, samples were quantified using a spectrophotometer (ND-1000, NanoDrop) and analysed by PCR followed by Sanger sequencing for *TAS2R38* polymorphism assessment. We compared the frequency of the alleles and of the genotypes in the different subgroups using the chi-square test (with Fisher correction); we considered a p value <0.01 to be significant.
